# Revealing the Sequence and Resulting Cellular Morphology of Receptor-Ligand Interactions during *Plasmodium falciparum* Invasion of Erythrocytes

**DOI:** 10.1371/journal.ppat.1004670

**Published:** 2015-02-27

**Authors:** Greta E. Weiss, Paul R. Gilson, Tana Taechalertpaisarn, Wai-Hong Tham, Nienke W. M. de Jong, Katherine L. Harvey, Freya J. I. Fowkes, Paul N. Barlow, Julian C. Rayner, Gavin J. Wright, Alan F. Cowman, Brendan S. Crabb

**Affiliations:** 1 Burnet Institute, Melbourne, Australia; 2 Department of Immunology, Monash University, Melbourne, Australia; 3 Department of Medical Biology, University of Melbourne, Australia; 4 The Walter & Eliza Hall Institute of Medical Research, Parkville, Australia; 5 Department of Microbiology & Immunology, University of Melbourne, Australia; 6 Centre for Molecular, Environmental, Genetic and Analytic Epidemiology, University of Melbourne, Australia; 7 Department of Epidemiology and Preventive Medicine and Department of Infectious Diseases, Monash University, Melbourne, Australia; 8 Schools of Chemistry and Biological Sciences, University of Edinburgh, Edinburgh, United Kingdom; 9 Malaria Programme, Wellcome Trust Sanger Institute, Cambridge, United Kingdom; 10 Cell Surface Signalling Laboratory, Wellcome Trust Sanger Institute, Cambridge, United Kingdom; MRC National Institute for Medical Research, United Kingdom

## Abstract

During blood stage *Plasmodium falciparum* infection, merozoites invade uninfected erythrocytes via a complex, multistep process involving a series of distinct receptor-ligand binding events. Understanding each element in this process increases the potential to block the parasite’s life cycle via drugs or vaccines. To investigate specific receptor-ligand interactions, they were systematically blocked using a combination of genetic deletion, enzymatic receptor cleavage and inhibition of binding via antibodies, peptides and small molecules, and the resulting temporal changes in invasion and morphological effects on erythrocytes were filmed using live cell imaging. Analysis of the videos have shown receptor-ligand interactions occur in the following sequence with the following cellular morphologies; 1) an early heparin-blockable interaction which weakly deforms the erythrocyte, 2) EBA and PfRh ligands which strongly deform the erythrocyte, a process dependant on the merozoite’s actin-myosin motor, 3) a PfRh5-basigin binding step which results in a pore or opening between parasite and host through which it appears small molecules and possibly invasion components can flow and 4) an AMA1–RON2 interaction that mediates tight junction formation, which acts as an anchor point for internalization. In addition to enhancing general knowledge of apicomplexan biology, this work provides a rational basis to combine sequentially acting merozoite vaccine candidates in a single multi-receptor-blocking vaccine.

## Author Summary

The development of an effective malaria vaccine is a world health priority and would be a critical step toward the control and eventual elimination of this disease. In addition, new pharmacological solutions are necessary as *Plasmodium falciparum*, the deadliest of the malaria-causing parasites, has developed resistance to every drug currently approved for treatment. Understanding the interactions required for the parasite to invade its erythrocyte host, as well as being valuable to our basic knowledge of parasite biology, is important for the development of drug-based therapies and vaccines. In this study we have, for the first time, filmed *P. falciparum* parasites invading erythrocytes while systematically blocking several specific interactions between the parasite and the erythrocyte. We have shown there is a sequential progression of specific interactions that occur in at least four distinct steps leading up to invasion. Previous vaccine attempts have targeted one or two of these steps, however, if a single vaccine were designed to block interactions at all four steps, the combined effect might so reduce invasion that parasite growth and disease progression would be arrested. A better understanding of each interaction during invasion, their role and order, can also inform the development of new anti-malarial drugs.

## Introduction

Malaria is caused by protozoan *Plasmodium* parasites and *Plasmodium falciparum* (*Pf*) is the most pathogenic of the five species known to infect humans, accounting for the majority of mortality from malaria. Recent clinical trials involving a pre-erythrocytic *Pf* vaccine, known as RTS,S, demonstrate partial efficacy [1,2], however, there remains a need to explore other vaccine options, especially those which have the potential of controlling blood stage infection. To prevent malaria caused by blood stage infection, pre-erythrocytic vaccines need to be capable of preventing virtually all parasites from exiting the liver to infect the blood. To date this has not been achieved, so pre-erythrocytic vaccines should therefore be paired with a blood stage vaccine to eliminate breakthrough parasites, thereby providing better protection from both clinical malaria and more severe sequelae. Vaccines targeting merozoites, the stage of the parasite that infects erythrocytes, have long shown promise, but their development has been hampered by limited functional knowledge of the molecular targets. In particular, while many receptor-ligand associations have been characterised, their distinct functions and relative contributions to invasion are not well established [3].

To improve our understanding of merozoite invasion, we filmed invasion of *Pf* merozoites and analysed the kinetics and morphology of its distinct steps [4,5]. We categorised these into three stages; pre-invasion, internalisation and echinocytosis, as was first described in *P. knowlesi* (Dvorak et al., 1975). The approximately 10 second pre-invasion step, is characterised by dramatic deformation of the target erythrocyte. Internalisation then ensues and 20–60 seconds later the newly infected erythrocyte takes on a stellate appearance, a phenomenon known as echinocytosis. The erythrocyte remains like this for 5–10 minutes before returning to its pre-invasion biconcave shape. The morphology and kinetics of these invasion steps are remarkably conserved across evolutionarily divergent *Plasmodium* species [4,5,6].

Despite its formidable technical challenges [7], live cell microscopy is a powerful tool for examining the behaviour of parasites and can reveal much about pathogenesis. Most studies of pharmacological or biological (i.e. antibodies) growth inhibitors of *Pf* consist of adding the inhibitor to parasite culture and measuring the parasitemia after a few days. This approach often provides little data on whether the inhibitor blocks growth, egress or invasion, and how quickly this occurs. While the effects of invasion inhibitors have been examined in great detail using fluorescent antibody probes or electron microscopy, it has usually been done with fixed cells, and therefore provides only a snapshot of a single moment in time during a rapid and highly dynamic process. To complement the considerable body of work on merozoite invasion using traditional microscopy methods with fixed cells, we have used live cell microscopy to provide an unprecedented examination of the process of invasion through systematically blocking ligands known to be involved. Here we have given new definition to these interactions, elucidating the resulting cellular morphology and temporal sequence.

A number of merozoite invasion ligands have been described. One group of these, merozoite surface proteins (MSPs), form a major component of the merozoite surface coat [3,8]. While there are many MSPs, the GPI-anchored merozoite surface protein 1 (MSP1) is both the largest, a dimer of >500kDa, and the most abundant [8,9,10,11]. Although the function of MSP1 remains unclear, the binding of exogenous heparin sulphate to MSP1 blocks invasion, suggesting it interacts with an as yet unknown erythrocyte receptor [12].

Another group of ligands involved in invasion are the ‘alternative-pathway’ ligands, so-called because the individual ligands appear to be functionally redundant. It is most likely, however, that these proteins have slight variation in their roles and work together with a combination of overlapping function and cooperation. In *Pf*, these ligands comprise the erythrocyte binding antigens (EBA-175, EBA-140, EBA-181 and EBL1), and the reticulocyte-binding like homologs (PfRh2a, PfRh2b and PfRh4) (reviewed in [13,14]). It appears that two PfRh proteins, PfRh1 and PfRh5, have distinct functions which are different from the others [15,16]. PfRh1 likely has a role immediately upstream of the alternative-pathway ligands, in signalling the release of micronemes containing EBA-175 [16], a process which is dependent on calcium [17]. While the alternative-pathway EBA and PfRh ligands bind to different erythrocyte receptors, studies with gene knockout parasites, in combination with mutant erythrocytes deficient in particular receptors, have shown that increased expression of some EBAs and PfRhs can functionally compensate for the lack of another [18,19,20]. This redundancy has most likely evolved to counter erythrocyte receptor polymorphism, although varying the expression of these ligands may also help the parasite circumvent the host’s antibody responses [21]. The other PfRh protein known to have a distinct function is PfRh5. Both the essentiality of PfRh5 [22], and the erythrocyte protein which PfRh5 interacts with, basigin [15,23], have been recently identified. Unlike other alternative ligands, PfRh5 lacks a transmembrane anchor, localises to the tight junction and appears anchored to the merozoite via the Ripr protein [15,24]. The PfRh5–basigin interaction, and hence invasion, can be blocked by antibodies to either protein but the precise role of this interaction in the process of invasion is unknown [15,22,25,26].

Once the merozoite binds to the erythrocyte, it reorientates its apical end onto the host cell surface and forms a connective ring between it and the erythrocyte. This ring is called the ‘tight junction’ or ‘moving junction’, and the merozoite passes through it to enter the erythrocyte powered by the parasite’s actin-myosin motor [27,28,29]. An important component of the tight junction is AMA1, a type 1 transmembrane protein, secreted onto the merozoite surface during egress from the old host cell. AMA1 binds to RON2, a member of the RON complex, which is translocated from the merozoite into the erythrocyte surface [27,30,31,32]. RON2 has an exposed loop that acts as the AMA1 binding site and in this way *Plasmodium* parasites encode their own ligand and host-embedded receptor. [29,33]. Both a synthetic peptide called R1 [34,35], and a peptide derived from the RON2 exposed loop, can block native RON2 binding by competing for the RON2-interacting groove of AMA1 and thus inhibit invasion [29,32,33,36]. Video microscopy of parasites treated with R1, or which have had their *ama1* gene deleted, shows that merozoites still bind to and deform erythrocytes, but they cannot properly invade and fail to progress to a ring stage [32,37,38]. This appears to be due to a defective tight junction that fails, either in assisting internalization, or in resealing of the host membrane behind the merozoite, in the few cases where internalization does occur [38].

**Fig 1:**
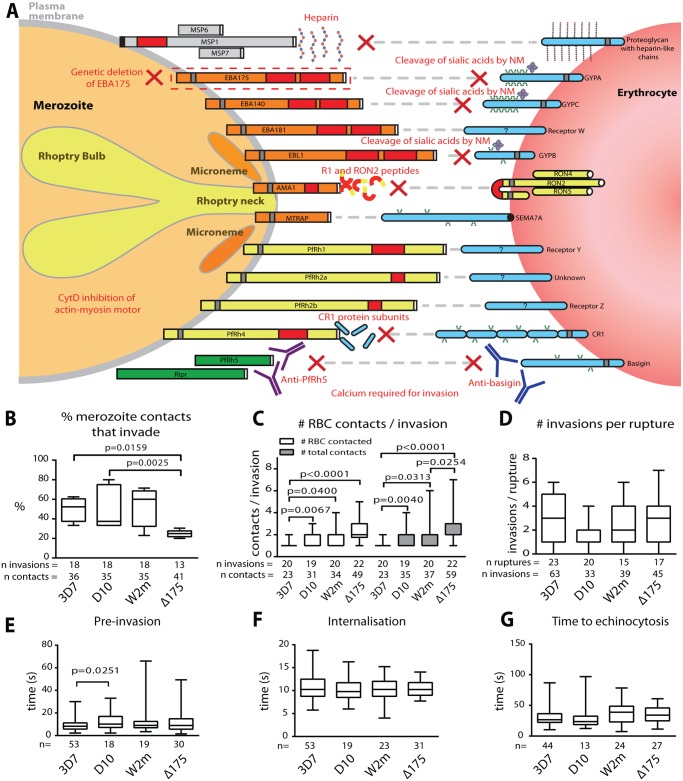
Merozoite invasion kinetics are generally similar for different *P. falciparum* strains. (A) Cartoon of merozoite ligands, their erythrocyte receptors, and methods used to ablate these interactions. (B-G) Box and whisker plots; line indicates median, box indicates 75–25% range, and ‘whiskers’ denote total data range. Significant differences (p<0.05) are shown on the graphs, differences approaching significance (0.05>p<0.1), and insignificant p values are noted in figure legends. The number of (n) invasions (B-G) and contacts (B-D) is shown below each graph. (B) Percent of merozoite—erythrocyte (RBC) contacts resulting in successful invasion is lower in Δ175 vs. other strains used in this study except for W2m (W2mef vs. Δ175 p = 0.151). Comparisons between all other strains were not significant (p = 0.621–0.891). (C) Both the number of erythrocytes contacted before merozoite invasion (white) and total erythrocyte contacts (as some were contacted more than once (grey)), do vary between strains. This approaches significance when comparing Δ175 vs. D10 (p = 0.062) and W2m (p = 0.052) in number of erythrocytes contacted, and Δ175 vs. D10 (p = 0.071) in total number of interactions. All other comparisons p = 0.605–0.764. (D) The average number of invasions per schizont rupture is nearly the same for all strains tested, however approaches a significant difference when comparing D10 vs. 3D7 (p = 0.056) and Δ175 (p = 0.055). All other comparisons p = 0.208–0.918. (E) The time from first contact of merozoite and erythrocyte to start of internalisation is nearly the same for all strains tested except when comparing D10 vs. 3D7. All other comparisons p = 0.107–0.960. (F) The time from start to finish of internalisation is nearly the same for all strains tested. All comparisons p = 0.592–0.970. (G) The time from finish of internalisation to start of echinocytosis is very similar for all strains tested. All comparisons p = 0.421–0.800.

At least a dozen receptor-ligand interactions are known to play roles in the invasion of erythrocytes by *Pf* merozoites and these are summarised in Fig. 1A. Here we have inhibited the following five receptor-ligand interactions using; 1) heparin sulphate to inhibit MSP1_42_ binding to unknown erythrocyte glycoproteins, 2) treatment with neuraminidase (NM) to remove sialic acids on glycophorin A (GYPA) and thus prevent binding of EBAs, 3) genetic deletion of EBA175 and NM treatment in addition to complement receptor 1 (CR1) fragments to block PfRh4 binding to CR1, 4) anti-PfRh5 and anti-basigin IgGs to block the binding of PfRh5 to basigin, and 5) R1 and RON2 peptides to inhibit AMA1–RON2 interactions.

To study these invasion pathways we have used live cell imaging while specifically ablating these interactions to characterise the role played by each receptor-ligand event in the morphological and physiological events that typify invasion. In addition to revealing the order of these events, we show that after initial contact, vigorous deformation associates with successful invasion. This deformation is mediated by the parasite’s actin-myosin motor and alternative-pathway receptor-ligand interactions. We further define the role of PfRh5, and provide evidence that an open connection forms between the apical tip of the parasite and the host cell immediately prior to invasion. We hypothesize that this open connection is mediated by PfRh5 to basigin binding and may act as a conduit for invasion proteins which establish the tight junction.

## Results

### The kinetics of invasion are conserved across different *Pf* strains

As a prelude to these invasion inhibition studies we first determined whether the four *Pf* strains, 3D7, D10, W2mef and ΔEBA175 (W2mef with its EBA175 gene deleted), used here, had similar invasion kinetics under permissive conditions. We began by examining the proportion of contacts between merozoites and erythrocytes that culminated in successful invasion. This method was chosen rather than total invasions per schizont rupture since it would remove some of the elements of chance such as differences in the density of target erythrocytes surrounding rupturing schizonts or the directions in which merozoites happened to be released. Putative brief contacts between merozoites and erythrocytes that produced no definitive deformation or adhesion period were discounted. For 3D7, D10 and W2mef, we observed that approximately half of parasite-host contacts progressed to invasion (Fig. 1B, S1 Table). In contrast, only 25% of the ΔEBA175 contacts resulted in invasion, however, this difference only reached statistical significance relative to 3D7 and D10.

Next, in a parallel approach, we counted the number of erythrocytes contacted before invasion occurred including the invaded erythrocyte. Both unique and total contacts (where the same erythrocyte may be contacted more than once) were counted (Fig. 1C). This indicated that 3D7 was the most efficient, since it contacted fewer erythrocytes before invading than did other strains (p≤0.04 for all comparisons), with 85% of 3D7 merozoites invading the first erythrocyte they encountered. On the other hand, ΔEBA175 was the least efficient tending to contact 2 erythrocytes on average before invasion although this was only significant relative to 3D7 and W2mef (Fig. 1C, S1 Table). Because ΔEBA175 does eventually invade, after more contacts, the number of invasions per rupture was comparable across all strains (Fig. 1D). Multiple schizont ruptures (n = 15–23) were observed for each strain to minimize discrepancies from rupture to rupture.

We also determined the periods of pre-invasion (primary contact, deformation and resting [5]), internalisation and the time to the commencement of echinocytosis and noted that, with the exception of 3D7 pre-invasion as compared to D10 (p = 0.025), there were no significant differences between the four strains tested (Fig. 1E-G). The mean lengths of pre-invasion ranged from 9–13 seconds, followed by 10–11 seconds for merozoite internalisation, which was the most tightly regulated phase of invasion with the narrowest range (Fig. 1E, F, S1 Table). From the completion of merozoite internalization to the beginning of erythrocyte echinocytosis, average times ranged from 31–38 seconds (Fig. 1G). Erythrocyte echinocytosis occurred following most, but not all, invasions (3D7: 76.7%; D10: 72.7%; W2mef: 91.9%; ΔEBA175: 83.8%). Examples of invasions for each strain are shown in S1–S4 Videos. As shown, the four strains used here have very similar invasion kinetics making it probable that the role of an invasion protein studied in one strain is conserved across all strains.

### Successful invasion correlates with preceding strong deformation

**Fig 2:**
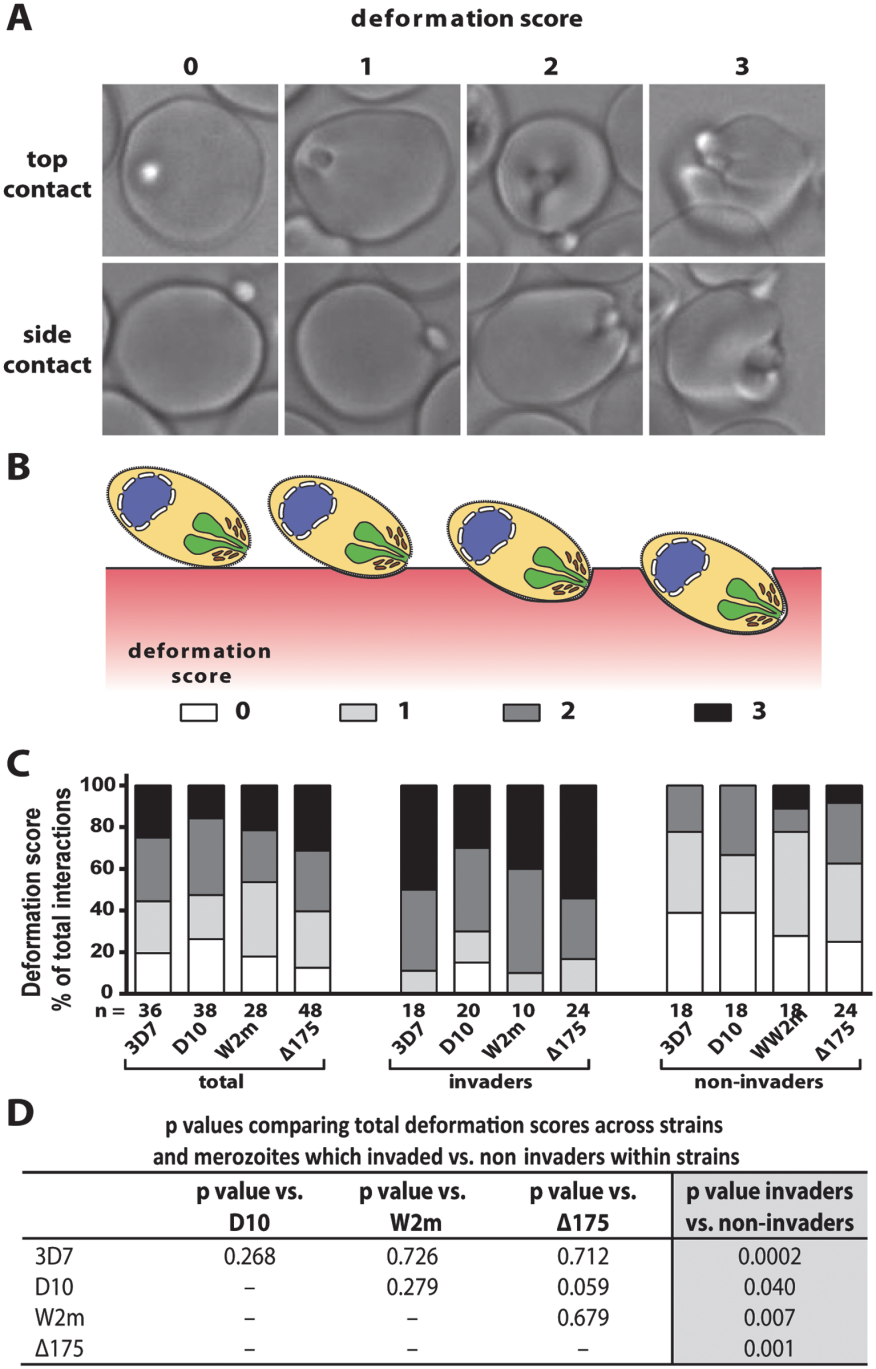
Strong merozoite deformation is associated with successful merozoite invasion. (A) Representative images demonstrating levels of merozoite deformation of erythrocytes. (B) Diagram illustrating the derivation of deformation scores. (C) The percentages of each deformation score for the parasite strains used. The total scores are shown first followed by breakdown into invading and non-invading merozoites. The number (n) of interactions scored for each strain is shown below the columns. (D) There is no difference in deformation scores between the strains used, however in all strains the invading merozoites deformed their target erythrocytes significantly more than non-invading merozoites (last column).

To further dissect the initial contact period we noted the degree to which merozoites deformed individual erythrocytes. Deformation is a complex occurrence that is difficult to quantify because it varies in intensity and time, lasting from a fraction of a second to several seconds and can occur in multiple waves. A simplified four-point deformation scale (0, 1, 2 and 3) was therefore devised, based on the most extreme degree of deformation achieved (Fig. 2A, B, and S1, S5–S7 Videos). When assessed using this scale there were no differences between the four parasite strains when all contacts were taken into account (Fig. 2C, D). However, there was a significant difference when comparing the deformation scores caused by merozoites which were invaders vs. non-invaders. The majority of merozoites that invaded deformed strongly (scores 2 and 3), while the majority of merozoites which did not invade deformed weakly or not at all (scores 0 and 1) (Fig. 2C, D). Thus, the degree of deformation correlated with the success of subsequent invasion.

**Fig 3:**
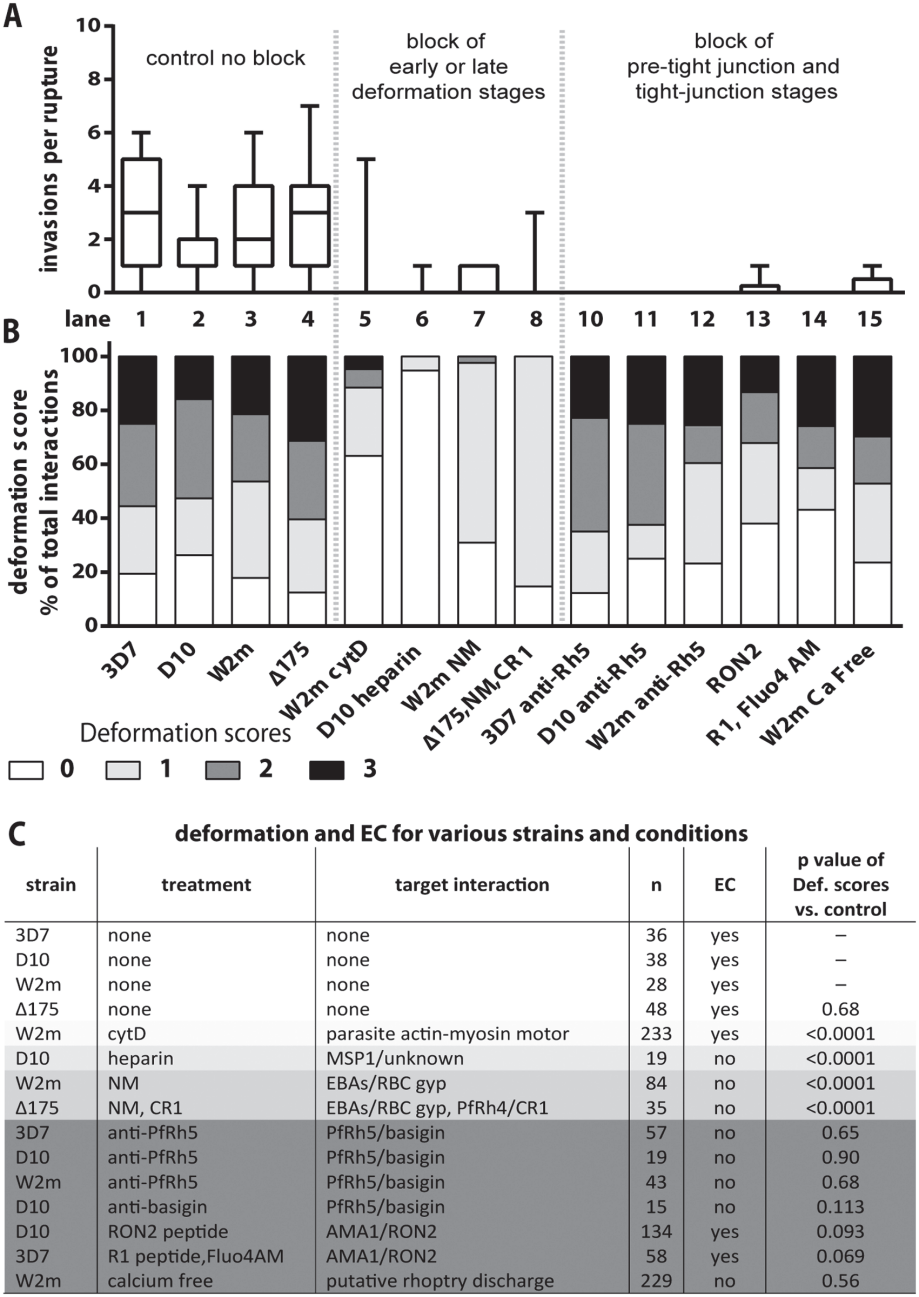
Blocking specific receptor-ligand interactions can decrease deformation, invasion and echinocytosis of the host erythrocyte. (A, B) Stacked graphs showing the number of invasions per rupture on top and matching deformation scores underneath. The parasite lines and treatments are shown at the bottom with untreated parasites in lanes 1–4 and parasites treated with invasion inhibitors in lanes 5–15. Note that data from 3C has been reproduced in lanes 1–4 of B in this figure, as controls for the invasion inhibitory treatments. (C) Table shows the parasite strain, the type of treatment, its target interaction, the number (n) of interactions counted, and whether echinocytosis (EC) of the contacted erythrocyte occurred. P values indicate whether the deformation scores for the treated parasites were significantly different (p<0.05) to untreated parasites. Note that Δ175 has a p value because it has been compared to the parental W2mef line.

To investigate the underlying causes of deformation we treated purified schizonts with cytochalasin D (cytD), an inhibitor of actin polymerisation, to block the merozoite’s actin-myosin invasion motor. The cytD not only blocked invasion as expected but also inhibited deformation with 88.4% of treated merozoites deforming their erythrocytes weakly or not at all (Def score 0–1), and only 11.6% deforming their erythrocytes strongly (W2m cytD, Lane 3 vs. 5, Fig. 3A,B, S2 Table, S8 Video). This data suggests that a functioning actin-myosin motor is required for deformation of the erythrocyte. To determine if there was any contribution made by the erythrocyte’s actin, we added untreated schizonts to erythrocytes treated with cytD and observed no differences as compared to untreated erythrocytes (S2 Table).

### Heparin does not prevent merozoite-erythrocyte contact but blocks deformation

It has previously been shown that heparin is capable of inhibiting an early step in invasion [12]. To quantify the effects we performed video microscopy of D10 parasite invasion in the presence of heparin and observed a 17-fold reduction in the number of invasions per schizont rupture (from 1.7 to 0.1, D10 vs. Heparin or Lane 2 vs. 6, Fig. 3A, S2 Table). In addition, heparin markedly reduced the capacity of merozoites to deform erythrocytes following contact and deformation scores of greater than 0 were rarely observed (Lane 6, Fig. 3B, S5 Video). In spite of dramatically reduced invasion and deformation, heparin treated merozoites maintained contact with erythrocytes for extended periods. Thus, a heparin-blocked protein is likely not the initial protein binding merozoites to erythrocytes, but is involved in an early binding event that mediates weak deformation, and blocking with heparin prevents both this weak deformation and progression to stronger levels of deformation. While heparin binds to several parasite proteins, the most obvious known candidate is the MSP1_42_ fragment of MSP1, which is already present on the merozoite surface at the time of egress and is thought to play a role early in invasion [12]. We hypothesize, therefore, that MSP1 may be responsible for mediating weak deformation of the erythrocyte, allowing progression to stronger deformation and subsequent invasion, and that blocking MSP1 via heparin prevents both this weak deformation and the progression to stronger deformation via downstream receptors.

### Alternative-pathway ligands mediate early contact events that are required for strong erythrocyte deformation

To examine the effect of blocking the alternative-pathway we used W2mef, a parasite strain that preferentially expresses EBA175 over other alternative-pathway ligands, and relies on it during invasion. The target erythrocytes were treated with NM to cleave sialic acid from erythrocyte glycoproteins and thus inhibit the EBA proteins from binding. With this block in place the invasion rate declined 9 fold from 2.6 to 0.3 invasions per rupture (W2m vs. W2m NM or Lane 3 vs. 7, Fig. 3A, S2 Table). While the majority of merozoites weakly deformed their erythrocytes, scoring 1, 2.4% of merozoite deformed more strongly, scoring 2 (Lane 7, Fig. 3B, C).

We further tested invasion in alternative pathway null conditions by filming ΔEBA175 merozoites which primarily use PfRh4, invading NM treated erythrocytes in the presence of soluble CR1 [39]. Under these alternative-pathway null conditions, the invasion rate declined 9 fold from 2.6 to 0.3 invasions per rupture, which is in line with the degree of inhibition previously published (W2m vs Δ175, NM, CR1 or Lane 4 vs. 8, Fig. 3A, S2 Table, S6 Video [20]). In terms of invasions per erythrocyte contact, this represents a decline from 22% to 4% (S2 Table). As the inhibited ΔEBA175 merozoites failed to deform strongly and the ratios of deformations scoring 0 and 1 are similar to W2mef under NM treatment, this suggests PfRh4 and EBA175 are performing comparable roles (Lane 7 vs. 8, Fig. 3B). Overall the data indicate that EBA/PfRh protein interactions most likely cause the strong deformation observed in the pre-invasion stage.

### Inhibition of PfRh5–basigin and AMA1–RON2 interactions block invasion even though merozoites still deform erythrocytes normally

To examine the role of PfRh5 binding to basigin we inhibited the interaction using rabbit anti-PfRh5 polyclonal IgG, which reduced invasion of 3D7 by ~90% [25]. For all parasite strains tested, a total of 42 schizont ruptures were filmed in the presence of anti-PfRh5 IgG with no successful invasions (3D7 vs 3D7 α-Rh5 or Lane 1 vs. 10, D10 vs D10 α-Rh5 or Lane 2 vs. 11 and W2m vs W2m α-Rh5 or Lane 3 vs. 12, Fig. 3A, S2 Table). Despite this, and in sharp contrast to the receptor-ligand events described above, the merozoites were able to bind and vigorously deform the erythrocytes to a similar degree as normal parasites (Lane 1–3 vs. 10–12, Fig. 3B, C, S9 Video). To determine if inhibitory anti-basigin antibodies would produce a similar effect to anti-PfRh5 antibodies, we observed invasions in the presence of the anti-basigin mAb MEM-M6/6 [22]. Similar to blocking with anti-PfRh5, in four schizont ruptures, no invasions were filmed compared to an average of 1.7 invasions per rupture without antibody (S2 Table). Likewise, although invasion was blocked, deformation with anti-basigin treatment was similar to that seen with anti-PfRh5 treatment, and deformation in both conditions was comparable to no treatment (Fig. 3C, S7 Video). This indicates that PfRh5 has a distinctive role from the other PfRhs and the EBAs, that appears to be downstream as the PfRh5-blocked merozoites can progress beyond weak to strong erythrocyte deformation. This putative role is further explored and substantiated by experiments shown below.

With PfRh5 appearing to function downstream of the EBA/PfRh ligands we next examined a step we hypothesized to be even further downstream, the AMA1 to RON complex interaction that forms the tight junction [29,30,32,33,36]. We filmed invading merozoites in the presence of RON2 peptide, which has been shown to block invasion with approximately 99% efficiency [36,40]. In our studies, treatment with RON2 peptide reduced the invasion rate of D10 merozoites approximately 8 fold, from 1.7 to 0.2 invasions per rupture (D10 vs RON2 or Lane 2 vs. 13, Fig. 3A, S2 Table, S10 Video). Some of the peptide treated merozoites appeared to embed themselves into the erythrocyte surface, however, they failed to transform into intracellular rings indicating invasion was unsuccessful. Deformation scores were similar to those of untreated parasites (Lane 2 vs. 13, Fig. 3B, C). Although the invasion-blocking effects of RON2 peptide appeared similar to that previously described for R1 peptide [40] we repeated this analysis here (Lane 14, Fig. 3A, S2 Table). We found the ratio of deformation scores in R1 peptide was similar to untreated parasites (Lane 1 vs. 14, Fig. 3B, C, S11 Video). Our data are consistent with AMA1 having a major function downstream of the EBA/PfRh’s, namely at the tight junction.

### The invasion motor is not required for reorientation and echinocytosis but for rapid host cell selection

**Fig 4:**
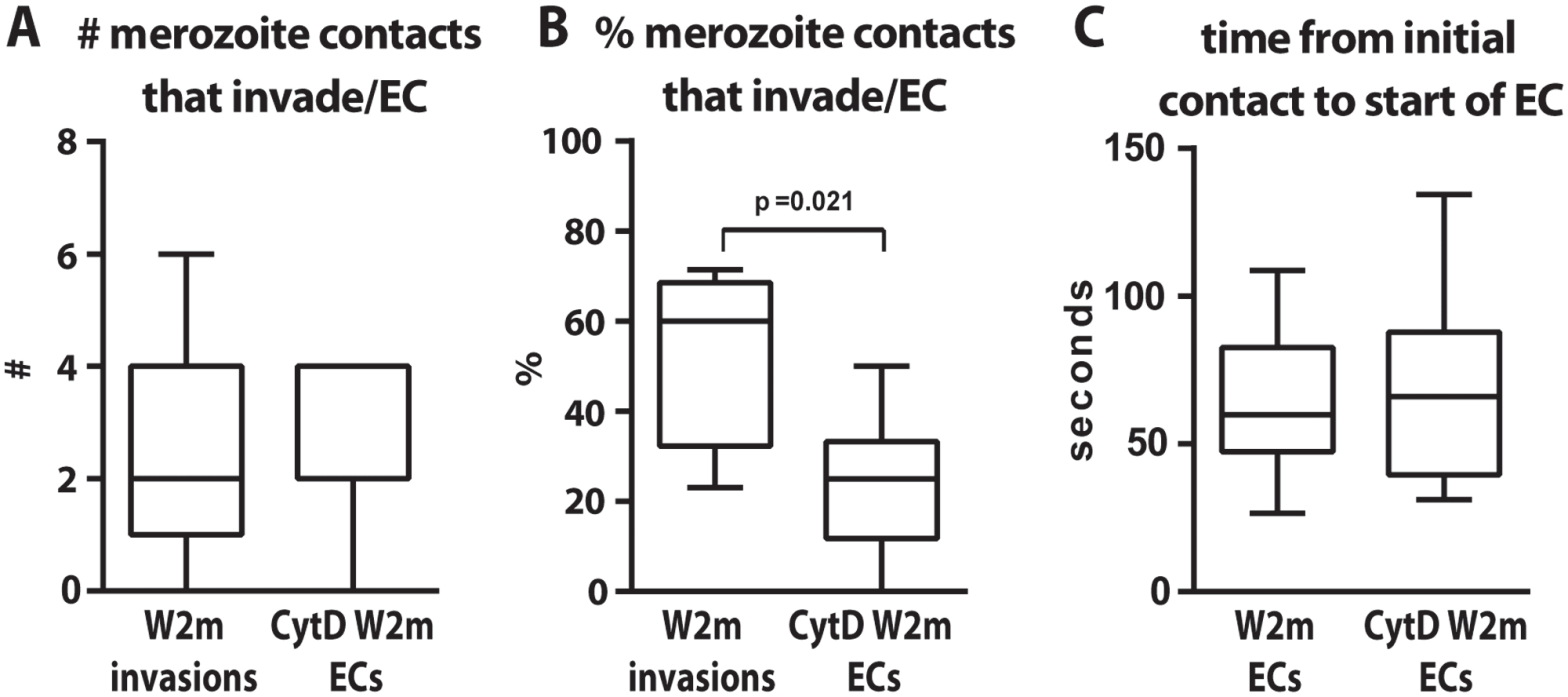
The parasite’s actin-myosin motor is involved in rapid host cell selection. (A) The number of merozoite contacts per schizont rupture which result in invasion in untreated parasites is the same as the number of merozoite contacts which result in echinocytosis in the invasion-blocked cytD treated parasites. (B) The percentage of merozoite contacts which result in invasion in untreated parasites and echinocytosis in the invasion-blocked cytD treated parasites. (C) The time from initial contact to the start of echinocytosis is shown for untreated vs. cytD treated parasites, irrespective of invasion.

Apart from merozoite invasion and deformation we also assessed whether the erythrocytes underwent echinocytosis after being contacted following the various inhibitory treatments. Merozoite contacts in heparin or following inhibition of the alternative pathways did not result in erythrocyte echinocytosis which is unsurprising since they were blocked quite early in the invasion sequence (Fig. 3C). Interestingly, cytD treated merozoites, despite being unable to deform and invade erythrocytes, were able to reorientate and trigger echinocytosis in the absence of invasion. A comparison between untreated and cytD treated W2mef parasites, revealed that per schizont rupture, there was no difference in the number of resulting invasions or echinocytes, respectively (Fig. 4A). However, cytD treated merozoites were less efficient at triggering echinocytosis in the first cell they contacted and tended to detach and contact additional erythrocytes before triggering echinocytosis in one of them (Fig. 4B). However, once an erythrocyte was selected, cytD-treated parasites caused echinocytosis within a similar time period as untreated parasites, irrespective of invasion (Fig. 4C). This indicates that the parasite’s actin-myosin motor is not required for reorientation and echinocytosis, but rather might be important for rapid host cell selection though deformation which possibly helps embed the merozoite in the erythrocyte surface, leading to subsequent downstream events.

### The PfRh5-basigin interaction occurs upstream of the AMA1–RON2 interaction

In cultures treated to block the PfRh5-basigin interaction, vigorous deformation did not result in echinocytosis (Fig. 3C, S7 and S9 Videos). When we blocked invasion by preventing the AMA1-RON2 tight junction interaction with RON2 or R1 peptides, echinocytosis occurred (Fig. 3C, S10 and S11 Videos). This places the AMA1-RON2 interaction downstream of PfRh5-basigin and suggests that echinocytosis is not caused by tight junction formation but rather an event upstream, possibly the PfRh5-basigin interaction. Rhoptry release from apically reorientated merozoites has previously been shown to occur even when the AMA1-RON2 interaction is blocked [27]. We thus hypothesize that the PfRh5-basigin interaction is responsible for triggering rhoptry release from apically reorientated merozoites, which in turn is responsible for stimulating echinocytosis, an event that can be separated from invasion.

### Absence of external calcium inhibits invasion similarly to blocking PfRh5

If erythrocyte echinocytosis is triggered by rhoptry release or some other perturbation to the erythrocyte membrane, what specific factor(s) is (are) causing echinocytosis? It had previously been hypothesised that entry of Ca^2+^ into the erythrocyte during invasion may elicit echinocytic shape changes by direct effects of elevated intracellular Ca^2+^ concentration [4] possibly by acting on the cytoskeletal mesh[41]. Since erythrocytes do not store Ca^2+^, an obvious Ca^2+^ source was the growth media and live cell imaging of invasion was therefore performed in Ca^2+^ free media to determine if echinocytosis still occurred. Prior to this, invasion assays were carried out with mature schizont cultures exposed to calcium containing RPMI and DMEM media and Ca^2+^ free DMEM media with or without EGTA over a 90 minute invasion window (S1A Fig.). These experiments indicated invasion was reduced several fold in Ca^2+^ free media in agreement with previous studies [42,43,44]. We then performed live cell imaging in Ca^2+^ free DMEM with EGTA and found a 13 fold decrease in the average number of invasions per schizont rupture relative to RPMI (S2 Table). Interestingly, without external Ca^2+^ the merozoites were still able to attach to and deform their target cells normally, but there was no echinocytosis, and thus the inhibition of invasion in Ca^2+^ free media occurred at the same point as it did when the Rh5-basigin interaction was blocked (Fig. 3, S2 Table, S1 Fig., S12 Video).

### Non-calcium rhoptry components appear to trigger echinocytosis

**Fig 5:**
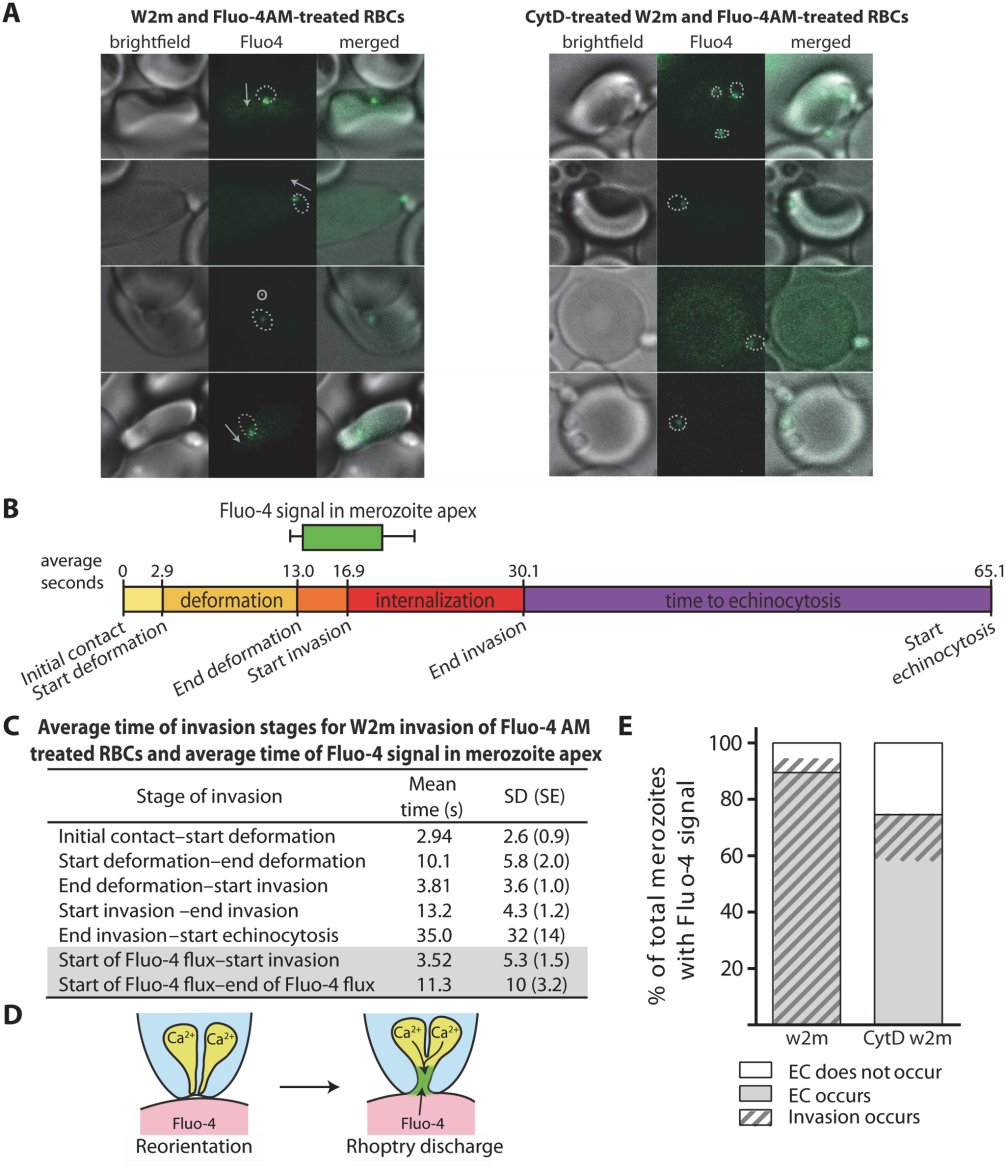
Immediately prior to invasion a Fluo-4 signal appears at the junction of the merozoite and host cell suggesting an opening between the erythrocyte cytoplasm and the merozoite. (A) Selected images from videos of purified W2mef schizonts added to Fluo-4 AM-labelled erythrocytes with (right) or without (left) cytD treatment. Outlines of the merozoite boundaries are transposed onto the Fluo-4 images to give an indication of where the Fluo-4 signal is relative to the merozoite. For those without cytD treatment, in the left panel, the direction of invasion is shown with an arrow, or a circle with a dot if the invasion is going into the plane of the image. (B) A timeline to scale showing the average length of time for each stage of the invasion process for untreated W2mef schizonts invading Fluo-4-treated erythrocytes. Above the timeline the average length of the Fluo-4 signal is indicated by the bar, with minimum and maximum shown by the whiskers, and placed on the timeline relative to the start of invasion. (C) Mean and SD for each stage of invasion and the timing of the punctate calcium signals observed for untreated W2mef schizonts invading Fluo-4-treated erythrocytes. (D) Model showing reorientation and illustrating how an open junction between merozoite rhoptries containing Ca^2+^ and erythrocyte cytoplasm containing Fluo-4 could mix at the merozoite apex, indicating a permeabilization or opening of the erythrocyte membrane. (E) Percent of total merozoites with punctate calcium signals which cause echinocytosis or do not, and within these the proportion which invade.

Having established that external Ca^2+^ was needed for invasion, we sought to determine if the external Ca^2+^ was entering the erythrocyte during invasion by visualising a potential flux with live cell imaging. We did this by purifying mature schizonts and adding them to erythrocytes labelled with the membrane permeable calcium-sensitive dye Fluo-4 AM. The majority of Ca^2+^ signals were punctate and confined to the invasion site (Fig. 5A, S13 Video). The punctate Ca^2+^ signal was detected 112 times in 248 invasions (45.2%). While there was approximately a half-second time lapse between brightfield and Fluo-4 images, allowing slight movement, it appeared that the punctate Ca^2+^ signal was located at the apical end of the merozoite (Fig. 5A). The Ca^2+^ signal first appeared near the end of deformation, on average 3.5 seconds prior to the initiation of invasion, and continued for an average of 11.3 seconds (Fig. 5B, C). Since rhoptry release into the erythrocyte surface has to precede invasion, the Ca^2+^ signal was being observed at around the same time we would expect the rhoptries to discharge. Occasionally, simultaneous to or immediately after the punctate Ca^2+^ signal, we observed a strong Ca^2+^ flux spreading into the erythrocyte from the invasion site prior to the start of echinocytosis (5.2% of invasions) (S14 Video). There were also occasional instances where a strong Ca^2+^ flux in the erythrocyte occurred during echinocytosis (3.4% of invasions). The intensity and magnitude of the signal suggested a large influx of Ca^2+^ was possibly coming from the media.

The punctate Fluo-4 fluxes could indicate that Ca^2+^ is a component of the rhoptry contents and that we are observing the ion being discharged into the host cell. The highly punctate and often confined nature of the signal could also indicate that Fluo-4 is entering the rhoptry compartment from the erythrocyte due to an opening forming in the erythrocyte membrane during rhoptry discharge (Fig. 5D). The punctate Ca^2+^ signal was also strongly associated with echinocytosis and invasion, with the Ca^2+^ signal observed in 59.2% of the cases where echinocytosis occurred. In addition, in 94.7% of cases where a Ca^2+^ signal was observed, echinocytosis followed, strongly linking the Ca^2+^ signal, and putative rhoptry discharge, to echinocytosis (Fig. 5E). However, the question remains; is it the Ca^2+^ or is it other components being discharged from the rhoptries such as proteins and lipids that cause echinocytosis?

**Fig 6:**
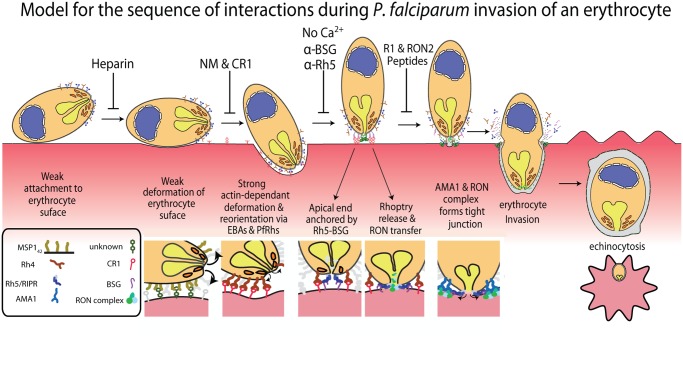
A new model for merozoite invasion. A diagram of an invading merozoite showing the order and function of the major receptor-ligand interactions studied here. Below this scheme are shown selected molecular interactions critical for invasion. This model incorporates for the first time a sequential hierarchy of distinct receptor-ligand steps during pre-invasion. We show the relationship of these interactions to erythrocyte deformation, which precedes normal successful invasion. Our data implies that rhoptry release is dependent on the interaction between PfRh5 and basigin, resulting in an opening in the erythrocyte membrane, and that external calcium is also important at this stage of invasion.

To investigate this further we performed live cell imaging of merozoites invading erythrocytes treated with BAPTA-AM to chelate rhoptry Ca^2+^ or even external Ca^2+^ leaking into the erythrocyte during invasion. Relative to untreated erythrocytes, no inhibition of deformation, invasion or echinocytosis was observed (S2 Table, S1B Fig.). The timing of all events leading up to invasion was also normal (S1C Fig.). Collectively our observations indicate that Ca^2+^ is probably not required for echinocytosis, and we hypothesize that upon rhoptry discharge other rhoptry contents enter the erythrocyte membrane and trigger echinocytosis independent of invasion (Figs 5 and 6).

In the absence of being able to directly visualise rhoptries discharging their contents we decided to use the punctate Ca^2+^ signal as marker for the release of these organelles and explore their putative role in triggering echinocytosis. It follows that if invasion was blocked under conditions where putative rhoptry Ca^2+^ signals were still observable, that echinocytosis should occur in the majority of cases. Conversely, when the rhoptry discharges were blocked, and no Ca^2+^ signals were observed, no echinocytosis should take place. The first invasion blocking condition tested where echinocytosis still occurred was with cytD-treated parasites. In this condition, apical orientation without deformation occurs, followed by tight junction formation but no erythrocyte internalization. In these parasites, we observed a punctate Ca^2+^ signal and where the alignment of merozoite and erythrocyte was side on, the Ca^2+^ signal localised to the region where the parasite and host made contact (Fig. 5A, right panel set). In 75% of cases where there was a Ca^2+^ signal, echinocytosis occurred (Fig. 5D), again reinforcing the link between the Ca^2+^ signal, echinocytosis and probable rhoptry release.

Similarly when we blocked with R1, which allows rhoptry release and echinocytosis, but not tight-junction formation or invasion, we observed the punctate Ca^2+^ signal which appeared at the same the time after merozoite contact as without R1, with the average signal starting at 16.3 seconds after contact in Fluo-4 alone and at 12.6 seconds after contact for Fluo-4 with R1. These R1 Ca^2+^ signals were observed in 39% of contacts that triggered echinocytosis (28 echinocytosis events and 11 Ca^2+^ signals, S11 Video). This further supports the use of the punctate Ca^2+^ signal as an indicator of rhoptry discharge which leads to echinocytosis.

Next, we attempted to verify that blockage of rhoptry release and subsequent echinocytosis would coincide with no observable punctate Ca^2+^ signals. Live imaging was performed on untreated schizonts with Fluo-4 AM treated erythrocytes in Ca^2+^ free media with EGTA, and of 11 schizont ruptures with 127 merozoite contacts we observed no Ca^2+^ signals (S15 Video, S2 Table). Next we imaged untreated schizonts with Fluo-4 AM treated erythrocytes in normal media containing anti-basigin IgG at 20, 10, and 2.5 μg/mL. In 20 μg/mL, from 22 schizont ruptures we observed no echinocytosis and 3 punctate Ca^2+^ signals in 128 merozoites that failed to invade their target erythrocytes but remained attached until the end of filming. In 10 μg/mL, from 19 ruptures we observed 2 incidents of echinocytosis (2.5%) and 9 punctate Ca^2+^ signals in 81 merozoites that failed to invade their target erythrocytes but remained attached until the end of filming. In 2.5 μg/mL, in 16 ruptures we observed 5 incidents of echinocytosis (5.9%) and 21 punctate Ca^2+^ signals in 85 merozoites that failed to invade their target erythrocytes but remained attached until the end of filming. This corresponds to punctate Ca^2+^ signals in 2.3%, 11.1%, and 24.7% of cases, respectively, and in comparison to 45.2% with no block, we see a dose-related increase in punctate Ca^2+^ signals with a decrease in anti-basigin antibody.

## Discussion

This study represents the first attempt to comprehensively overlay known receptor-ligand interactions with the morphological effects on erythrocytes and physiological events that occur in the seconds preceding, during and immediately after merozoite invasion of erythrocytes. By linking receptors to morphological effects on erythrocytes, as well as physiological and kinetic features, we show the probable order of sequentially acting receptor-ligand interactions leading up to invasion. In order, these are a heparin-blocked interaction (possibly MSP1 binding an unknown erythrocyte receptor [12]), the alternative-pathway PfRh/EBA ligands binding a range of known and unknown host receptors, PfRh5 ligand binding the erythrocyte basigin receptor and finally the AMA1 ligand binding to another parasite protein, RON2, that has been translocated into the erythrocyte membrane (Fig. 6) [22,30,39].

To investigate the functional order of invasion ligands, three parasite strains and one mutant derivative were characterized. Strain 3D7 was chosen because inhibition of invasion in this strain has previously been characterized with a variety of laboratory reagents including R1 peptide. Recently, the D10 strain has gained favour because its 48-hour cell cycle permits ease of use relative to other lines. To study EBA/PfRh function, the EBA175 dominant line, W2mef, was chosen since this ligand is easily blocked by NM treatment. A derivative of this line, the ΔEBA175 mutant was also assayed because its switch to dominant use of the CR1-dependent PfRh4 ligand can be blocked with soluble CR1 subunits. Before commencing our study of inhibition of invasion it was important to ensure the lines invaded with similar frequencies and kinetics, which they did. The exception was in the number of invasions per erythrocyte contact, where merozoites lacking EBA175 were only half as efficient as other strains tested. ΔEBA175 parasites also interacted with more erythrocytes prior to invasion than did other strains, thus overall invasion rates were comparable. In most parasites EBA175 is a dominant ligand with about a million copies of its receptor, glycophorin A, available for binding (reviewed in [45]). In contrast, there are only about a thousand molecules per erythrocyte of CR1, the receptor for PfRh4 and the dominant ligand in ΔEBA175 parasites. The relatively low number of CR1 molecules could result in lower avidity, with the parasites detaching more readily, both preventing invasion and allowing merozoites which are still viable to interact with other erythrocytes. Thus, both the reduction in efficiency of invasions per contact in ΔEBA175 parasites, and the increased number of erythrocytes contacted prior to invasion could be due to the abundance of the respective erythrocyte receptors. The difference in receptor abundance, however, does not translate into different growth rates between the ΔEBA175 mutant and its W2mef parent strain, both in our data presented here and in unpublished growth rate data (Lopatiki and Cowman, see Acknowledgments).

Of the receptor-ligand interactions that we inhibited, MSP1 and the EBAs/PfRhs reduced the ability of merozoites to deform their target erythrocytes and invade. In addition, cytD treatment of parasites, but not erythrocytes, inhibited deformation and invasion, indicating that function of the actin-myosin motor is also required to deform the erythrocyte during apical reorientation prior to the motor’s role in host cell internalization. MSP1_42_ is a target of heparin [12], which almost completely ablated deformation. This block of progression to stronger deformation suggests both that MSP1_42_ binding is responsible for weak deformation and that this leads to stronger deformation, reorientation and invasion. Although heparin is known to bind to other invasion ligands [46], because it inhibits an early step in invasion, MSP1_42_ could be the main invasion blocking target of heparin [12]. Specific inhibition of MSP1 using an antibody could help validate its role in early deformation, but unfortunately, the only known MSP1 invasion inhibitory antibody blocks surface shedding and therefore would be expected to function further downstream as the merozoite passes through the tight junction [47]. Heparin did not stop merozoites binding to erythrocytes suggesting other ligands are responsible for primary attachment.

The weak deformation (scoring 1) mediated by MSP1 could be passive in nature, as GPI-anchored proteins such as MSP1, have fluid movement in membranes and, as they aggregate at the site of merozoite contact, could form a depression in the erythrocyte membrane. This “cap” of aggregated GPI-anchored ligands could hold the merozoite to the erythrocyte membrane but still allow the merozoite to rotate and move until the EBAs and PfRhs bind their respective partners. Blocking EBA175 and PfRh4 strongly inhibited both deformation and invasion. The observation that both EBA175 and PfRh4 function similarly, supports a long held view that these and most of the other EBA/PfRh ligands have redundant functions [48]. This, however, likely does not apply to all of these ligands since PfRh1 binding was recently shown to trigger parasite calcium signalling and EBA175 release [16], and a separate role for PfRh5, late in invasion, has been indicated by data presented here.

Strong deformation (scoring 2 and 3) that culminated in apical reorientation could be produced passively by a gradient of high affinity EBAs/PfRhs emanating from the merozoite apex or could be actively powered by the parasite’s actin-myosin motor interacting with the cytoplasmic tails of the EBAs/PfRhs. To discriminate between these we blocked the actin-myosin motor with cytD and the merozoites no longer deformed strongly, suggesting a role for the motor in powering deformation in addition to its role in internalization. Unexpectedly, the cytD treated merozoites could reorientate their apical ends onto the erythrocyte surface, release their rhoptries and presumably form a tight junction and trigger echinocytosis in the same time frame as untreated parasites. This indicates a gradient of EBA and PfRh proteins are passively responsible for reorientation and so what then is the function of strong deformation? We observed in cytD treated merozoites that they contacted more erythrocytes before finally selecting one with which to form a tight junction than did untreated parasites. Could the actin-myosin powered movement of EBA and PfRh molecules be causing deformation to help to embed the merozoite into the erythrocyte surface so it is less easily detached and more rapidly commits to invasion? Within an *in vivo* setting, strong binding, embedding into the host cell, and rapid host cell selection could be important to out-pace host immune responses and the shear forces associated with circulation.

Whilst attempting to determine the sequence of invasion events, some key new findings have emerged. We show that successful invasion correlates with preceding strong deformation suggesting that vigorous surface contacts might have a function in promoting invasion or triggering downstream signalling required for subsequent invasion steps. Variability in the time and amplitude of deformation may correspond to where the merozoite makes initial contact with its target erythrocyte. For example, if the merozoite contacts near its apical end, then shorter and shallower deformation may occur prior to apical reorientation than if the merozoite makes contact with its basal end. To validate this would require the apical end to be visibly discernible from the rest of the merozoite during reorientation, but unfortunately we could not distinguish this using brightfield illumination alone. The apical end was, however, evident with Ca^2+^ imaging after reorientation where an apical Ca^2+^ signal was observed.

When Fluo-4 AM dye was loaded into erythrocytes and added to unstained parasites, we observed a Fluo-4 flux at the apical end of the merozoite immediately prior to invasion. As erythrocytes have no known Ca^2+^ stores and the parasites were not labelled with Fluo-4 AM, this suggests that a Ca^2+^-containing organelle in the parasite is coming into contact with contents of the erythrocyte or that Ca^2+^ from the media is entering possibly via a pore. Previous observations indicate that rhoptry release still occurs when the AMA1–RON2 interaction is blocked [27] and here we show that when the AMA1–RON2 interaction is inhibited, the Ca^2+^ signal in the parasite still occurs. Further, when the parasite’s actin-myosin motor was blocked with cytD, the Ca^2+^ signal was still observed. Presence of the Ca^2+^ signal associates strongly with echinocytosis in untreated parasites as well as cytD and RON2–treated parasites. Together, this implicates that the rhoptries release factors that result in permeabilization of the host cell, Ca^2+^ entry and the triggering of echinocytosis.

That Ca^2+^ entry might be triggering echinocytosis by changing the cytoskeletal mesh was tested by performing invasions in BAPTA-AM treated erythrocytes to chelate the ion upon entry. Chelation of Ca^2+^ had little effect upon any aspect of invasion apart from slightly decreasing the time to echinocytosis contrary to expectations. Since the secretion of lipid-rich rhoptry contents have been observed to penetrate the host membrane and form whorls within its cytoplasm, even when invasion is blocked with cytD [49,50], these lipids could be responsible for echinocytosis. It has been proposed that materials which insert into the outer leaflet of a cell membrane will cause outward bending, leading to crenation of the cell [51]. The released rhoptry lipids would first make contact with the outer leaflet of the erythrocyte before penetrating as whorls into the cytoplasm. The excess material in the outer leaflet could cause transient crenation and echinocytosis of the host cell before flippases correct the asymmetry and return the echinocyte to its biconcave shape after several minutes, which is what we observe [38]. In RON2 and R1 blocked invasions we often observe that echinocytes fail to quickly return to their pre-invasion shape, even after 30 minutes which was the length of the filming period. It has been noted previously that in the presence of R1 peptide, rhoptry material was distributed over the outside of the erythrocyte membrane rather the penetrating it, since it was not confined within the boundary of the tight junction [27]. The deposition of the rhoptry’s full contents into the outer leaflet of the erythrocyte membrane could trigger great imbalance in bilayer components, which is why it could take longer for the erythrocyte to recover its normal shape.

One of our most interesting new findings was that when PfRh5–basigin was blocked, or when invasion was inhibited by absence of extracellular calcium, incidences of echinocytosis virtually stopped, suggesting that the rhoptries were not released. The fact that an apical Ca^2+^ signal was rarely observed in Fluo-4 labelled erythrocytes when the PfRh5-basigin interaction was blocked corroborates this and suggests that PfRh5–basigin binding mediates a pre-tight junction in a calcium-dependant manner that triggers rhoptry release. Calcium does not appear to be required for PfRh5 binding to basigin [52] and it may be required for some upstream or downstream function. As external calcium is required for proteases namely PfSUB2, that shed the merozoite surface coat [53] [54], we did not initially suspect rhoptry release would be affected. Discharge of the rhoptries releases the RON complex that then crosses to the erythrocyte surface in order to form a tight junction with AMA1 to allow subsequent invasion of the erythrocyte.

The Fluo-4 signal observed prior to and during invasion could be due to release of Ca^2+^ from the rhoptries and/or diffusion of erythrocyte Fluo-4 into the rhoptry neck region via a pore. Alternatively, media Ca^2+^ could be entering the erythrocyte at the invasion site. To discriminate between these, we tested for Fluo-4 signals in Ca^2+^ free media with the expectation that if a signal was observed it must be derived from the merozoite, or if absent, then the Ca^2+^ must be from media. The result however proved to be inconclusive for although no Fluo-4 signal was observed there appeared to be no release of rhoptries (because there was no echinocytosis) that would have provided a putative pore for entry of Ca^2+^ into the erythrocyte.

Recently it was reported that *P. berghei* parasites with their *ama1* gene deleted grow at about a third the rate of wildtype parasites and can still penetrate their target erythrocytes and form a parasitophorous vacuole membrane (PVM) [55]. We concur with the latter because we have observed here that a few RON2 peptide treated merozoites still penetrated their host cells. Previously, we also observed penetration in AMA1 knock down *Pf* merozoites, however in both cases we did not observe merozoites transforming into rings after internalization, and there was a delay in the recovery of normal erythrocyte shape after echinocytosis [38]. We suspect, therefore, that there was a defect in erythrocyte resealing at the invasion site, which could be one of the additional functions of the AMA1-RON2 tight junction. Normal PVM formation in *P. berghei* Δ*ama1* parasites is compatible with our own observations and those of others, namely that AMA1 function is not needed for the release of rhoptry contents to form the PVM [27,55].

Live cell imaging can reveal much about parasite behaviour and pathogenesis. Our work here sheds light on the specific roles and order of receptor-ligand interactions leading up to invasion, expanding our knowledge of the biology of Plasmodium. This work also lays the foundation for the development of a sequential-step invasion-blocking vaccine, which might provide a significant biological advantage by targeting proteins functioning at multiple sequential steps of invasion, rather than targeting multiple proteins that function at the same step.

## Materials and Methods

### Parasite culture

*Pf* strains 3D7, D10 PfM3’ [56], W2mef and W2mefΔEBA175 were maintained in continuous culture as per [57]. Where indicated, parasites were also cultured in Dulbecco’s Modified Eagle Medium (DMEM, Gibco®) supplemented with L-glutamine (Sigma) and Albumax II (Invitrogen). Parasites were synchronised using sorbitol and heparin treatments as described previously [58].

### Live-cell microscopy

Highly synchronous parasite cultures at 4% hematocrit were diluted to 0.16% in RPMI media or washed in Ca^2+^ free DMEM+EGTA media (see below) and 2 mL of this was allowed to settle to produce a monolayer onto a 35 mm Fluorodish (World Precision Instruments). Custom dishes holding smaller 25 or 50 μL volumes were used for experiments containing invasion inhibitory antibodies. All live-cell experiments were performed at 37°C on a Zeiss AxioObserver Z1 fluorescence microscope equipped with humidified gas chamber (90% N_2_, 1% O_2_, and 5% CO_2_). Late stage schizonts were observed until they looked ready to rupture (described in [7]) and time-lapse videos were recorded with a AxioCam MRm camera usually at 4 frames per second. ImageJ and Prism (Graphpad) were used to perform image and statistical analyses. For data sets with normal distribution (Figs 1E-G, 4C, and S14C) an unpaired t test was used. For data sets without normal distribution (Figs 1B-D, 3A, 4A&B, S6 and S14A&B) the Mann-Whitney test was used. For comparison of deformation scores between groups (Figs 2, 3B&C and 5E) a Chi-square analysis was performed. A value of p≤0.05 was used as the determinant of statistical significance for all tests.

### Scoring

To score the degree of merozoite deformation of the erythrocyte surface we initially attempted to develop software to automate the process but were unable to. Instead, we resorted to scoring deformation by eye using a simplified deformation score and multiple trained scorers. We defined contact between the merozoite and erythrocyte as interactions where the erythrocyte and merozoite maintained physical contact with each other for two frames (0.25 seconds) or longer. The vast majority of contacts which we observed lasted considerably longer than 0.25 seconds. Interactions where the merozoite and erythrocyte appeared to touch for a single frame were not counted unless there was evident deformation, indicating clear contact, however these were very rare events. A deformation score of: 0 = sustained contact but no deformation, 1 = weak deformation at the point of contact, 2 = strong deformation with the erythrocyte membrane extending up sides of the embedded merozoite, and effects of deformation no longer strictly local to the merozoite, and 3 = extreme deformation with the deeply embedded merozoite partially covered by the erythrocyte membrane and extremely strong deformation distant from the merozoite to the point of distortion of erythrocyte borders (Fig. 2A, B).

### Reagents

Invasion-blocking reagents were used as follows; Heparin 100 μg/ml (Sigma), 100 μg/mL R1 peptide (Mimotopes) [35], 10 μg/mL RON2 peptide (LifeTein LLC) [36], 1 μg/mL cytochalasin D (Sigma), 10 μg/mL anti-basigin mAb MEM-M6/6 [22], and 10 mg/mL rabbit anti-PfRh5 [25]. Calcium within erythrocytes was chelated using BAPTA-AM (Sigma). The stock BAPTA-AM prepared in DMSO was added to erythrocytes at 0.16% hematocrit to achieve the final concentration of 50 μg/mL. The treated erythrocytes were incubated at 37° for 30 min, and then were washed three times. To inhibit merozoite invasion in W2mef ΔEBA175 parasites 0.2U/mL neuriminidase (Sigma) was added directly to the parasitized erythrocytes for 30 mins at 37°C and 10 μg of CR1 CCP1–3 was added prior to microscopy [20,39].

### In-vitro calcium invasion assays

Control invasion assays were performed in complete RPMI or DMEM media (Life Technologies). For Ca^2+^ free experiments Ca^2+^ free DMEM was used (Life Technologies), both in its standard form and modified with 2.5 mM EGTA since Ca^2+^ free RPMI was not commercially available. The media were supplemented with 25 mM HEPES and dialysed calcium free Albumax II to 0.5% and prior to use all the media were supplemented with 0.2% NaHCO_3_. The invasion experiments were conducted with two transfected 3D7 parasites lines expressing secreted or exported Nanoluciferase [59]. Late stage magnet purified schizonts (~2 × 10^8^) were split into four equal portions and each was washed in 1000 volumes of either RPMI, DMEM, Ca^2+^ free DMEM or Ca^2+^ free DMEM+EGTA. In parallel, uninfected erythrocytes were washed the same media types and then each batch of parasites and erythrocytes of the same media type were mixed together to final hematocrit of 2%. Each of the fractions was then split into two and heparin (Sigma) at a final concentration of 100 μg/mL was added to one half of the parasites to block invasion (Boyle et al 2010). The parasites were then incubated at 37°C for 90 mins to permit some invasion to occur. The infected erythrocytes were then pelleted at 3000g and a small amount of media retained to measure luciferase activity to ensure that egress and Nanoluciferase release had occurred. The parasites treated with 5% sorbitol to lyse the remaining schizonts and merozoites. The sorbitol was then replaced with complete RPMI media and the new ring stage parasites were grown in triplicate 100 μL cultures in a 96 well plate for 24 and 72 hrs to amplify the invasion signal. To assay parasite growth at 2, 24 and 72 hrs post invasion, 5 μL of the culture was lysed in 50 μL of water containing 1/1000 NanoGlo substrate (Promega). Relative light units (RLU) were measured in a FLUOstar Omega Luminometer (BMG Labtech) for 2s with the gain set to the brightest well and adjusted to 10% below saturation. To derive the invasion signal, the RLU from the heparin blocked invasion samples were deduced from their corresponding heparin free samples.

### Calcium imaging

Fluo-4 AM (Life Technologies) was added to a final concentration of 10 μM to erythrocytes diluted in incomplete RPMI with no albumax or serum at 2% hematacrit. The erythrocytes were incubated at 37° for 1 hr and washed 3x in 10 volumes in complete RPMI or Ca^2+^ free DMEM+EGTA before adding to a 35 mm Fluorodish as described above. Magnet purified late schizont stage parasites was added to the dish. The parasites were imaged with low power brightfield conditions (2–3 volts) until rupture occurred whereupon alternative brightfield and fluorescence images with the GFP filter set were recorded. To limit photodamage each 35 mm dish was only imaged four times, once per quarter, and the 50 μL dishes were prepared fresh for each movie made.

## Supporting Information

S1 Table Corresponding to Fig. 1 panels B-G either the *median and interquartile range (IQR) (in italics)* for non-normal distributions, or mean and standard deviation (SD) for normal data distributions is shown. Mz, merozoites.
(TIF)

S2 Table Table showing the number of invasions per rupture for various strains and conditions. Shown are the number of invasions by mean and median, the p value vs. the respective control for each condition, and the percent of invasions per erythrocyte (RBC) contact.
(TIF)

S1 Fig Merozoite invasions in modified calcium conditions. (A) Invasion assay in normal (RPMI and DMEM) and calcium free (DMEM no Ca^2+^ and DMEM No Ca^2+^ +EGTA) media indicate that merozoites need calcium for efficient invasion. Late schizont-stage parasites expressing Nanoluciferase were incubated with erythrocytes in the various media for 90 minutes-/+ heparin at 100μg/mL. After this the unruptured schizonts and merozoites were destroyed by sorbitol treatment and the new ring-stage parasites resulting from successful invasions were grown were for 72 hrs in complete RPMI media. The quantity of parasites was measured by assaying their luciferase levels in relative light units (RLU). To remove background levels of breakthough parasites ie, those parasites that had already invaded prior to the start of the experiment or had not been removed by sorbitol treatment, the RLU of heparin was subtracted from the untreated RLU. The median RLU of DMEM was normalised to 1.0 and the RLU of the other media relative to this are shown. The data represent four separate experiments each performed with triplicate samples. (B) Video microscopy of untreated parasites (W2m) and parasites whose erythrocyte hosts had been treated with BAPTA-AM indicate that chelation of Ca^2+^ introduced during invasion did not reduce the invasion rate in terms of invasion per schizont rupture. In contrast, treatment with Ca^2+^ free media +EGTA, caused the number of invasions per schizont rupture to significantly decline. (C) Comparison between the timings of W2mef invasion steps into BAPTA-AM treated and untreated erythrocytes indicates chelation of erythrocyte calcium has no major impact except for slightly decreasing the time to echinocytosis. For all figures, the horizontal line denotes the median, the box denotes the 25^th^ to 75^th^ percentiles and the whiskers the total data range.
(TIF)

S1 Video 3D7 merozoites invading erythrocytes (4 fps). Invading merozoites are indicated by different colored arrows. Label abbreviations: Con, Contact; Def S, Deformation start; Def n, Deformation score n; Inv S or E, Invasion Start or End; EC, Echinocytosis and Ring, Ring stage parasite.
(MP4)

S2 Video D10 PfM3’ merozoites invading erythrocytes (4 fps). (MP4)

S3 Video W2mef merozoites invading erythrocytes (4 fps). (MP4)

S4 Video W2mef ΔEBA175 merozoites invading erythrocytes (4 fps). (MP4)

S5 Video D10 merozoites attempting to invade erythrocytes in the presence of 100μg/ml heparin sulphate (4 fps, 2x real speed). (MP4)

S6 Video W2mef ΔEBA175 merozoites attempting to invade neuraminadase-treated erythrocytes in the presence of CR1 protein CCP1–3 fragment (4 fps). (MP4)

S7 Video D10 PfM3’ merozoites attempting to invade erythrocytes in the presence of anti-basigin mouse monoclonal antibody (2 fps; 2× real speed). (MP4)

S8 Video CytD treated W2mef merozoites attempting to invade untreated erythrocytes (4fps). (MP4)

S9 Video D10 PfM3’ merozoites attempting to invade erythrocytes in the presence of rabbit anti-PfRh5 polyclonal IgG (4 fps; 2× real speed). (MP4)

S10 Video D10 PfM3’ merozoites attempting to invade erythrocytes in the presence of RON2 peptide (2 fps; 10× real speed). (MP4)

S11 Video Fluo-4-stained 3D7 parasite culture showing merozoites attempting to invade in the presence of R1 peptide (variable speed). Brightfield and fluorescence images were sequentially captured.
(MP4)

S12 Video W2mef parasites in calcium free DMEM media +EGTA attempting to invade erythrocytes (4 fps). (MP4)

S13 Video Fluo-4 AM stained erythrocytes being invaded by W2mef merozoites showing punctate apical calcium flux (0.5 fps). Brightfield and fluorescence images were sequentially captured.
(MP4)

S14 Video Fluo-4 AM stained erythrocytes being invaded by 3D7 merozoites showing punctate apical calcium flux that spreads into the entire erythrocyte. Brightfield and fluorescence images were sequentially captured.
(MP4)

S15 Video Fluo-4 AM-stained erythrocytes invaded by W2mef merozoites in the presence of calcium free DMEM media with 2.5 mM EGTA. Brightfield and fluorescence images were sequentially captured.
(MP4)
