# Supplementary figures and images for: Revealing the Sequence and Resulting Cellular Morphology of Receptor-Ligand Interactions during Plasmodium falciparum Invasion of Erythrocytes
Source: PLoS Pathog. 2015 Feb 27;11(2):e1004670. doi: 10.1371/journal.ppat.1004670 (PMC4344246; doi:10.1371/journal.ppat.1004670)

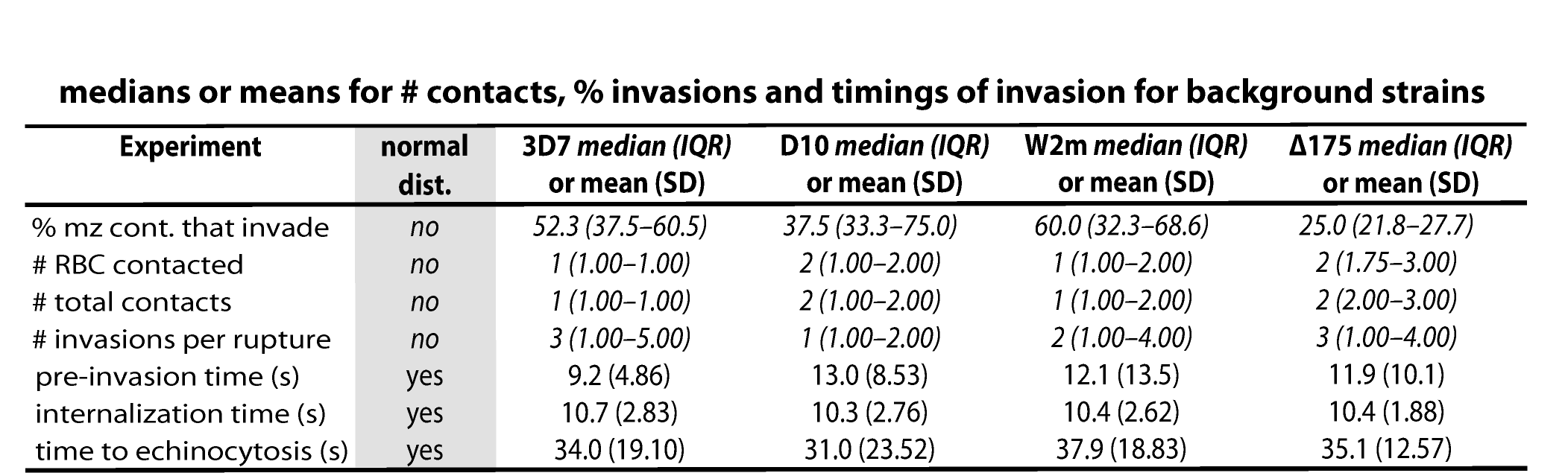

Supplement: S1 Table — Mz, merozoites. (TIF) [file ppat.1004670.s001.tif]

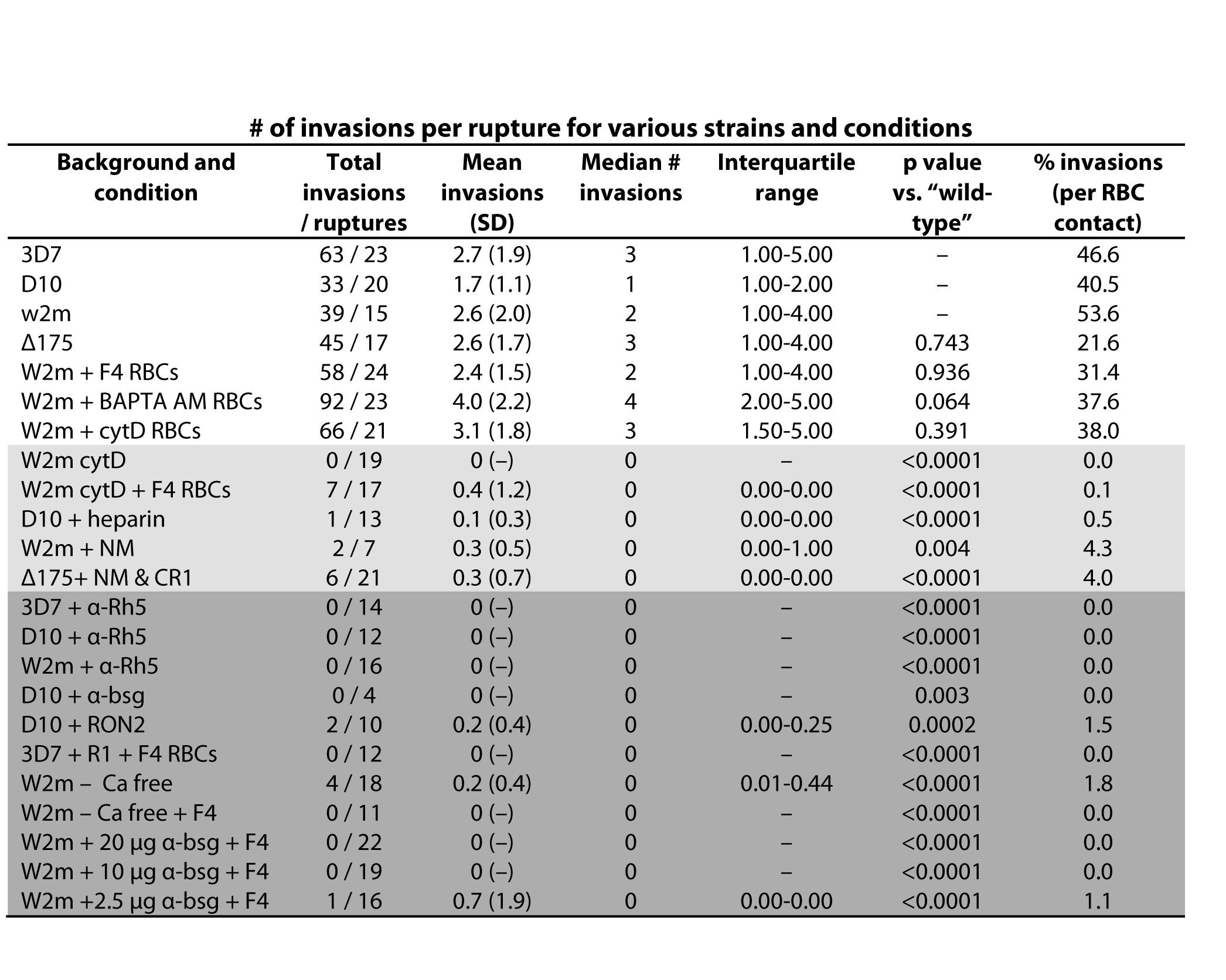

Supplement: S2 Table — Shown are the number of invasions by mean and median, the p value vs. the respective control for each condition, and the percent of invasions per erythrocyte (RBC) contact. (TIF) [file ppat.1004670.s002.tif]

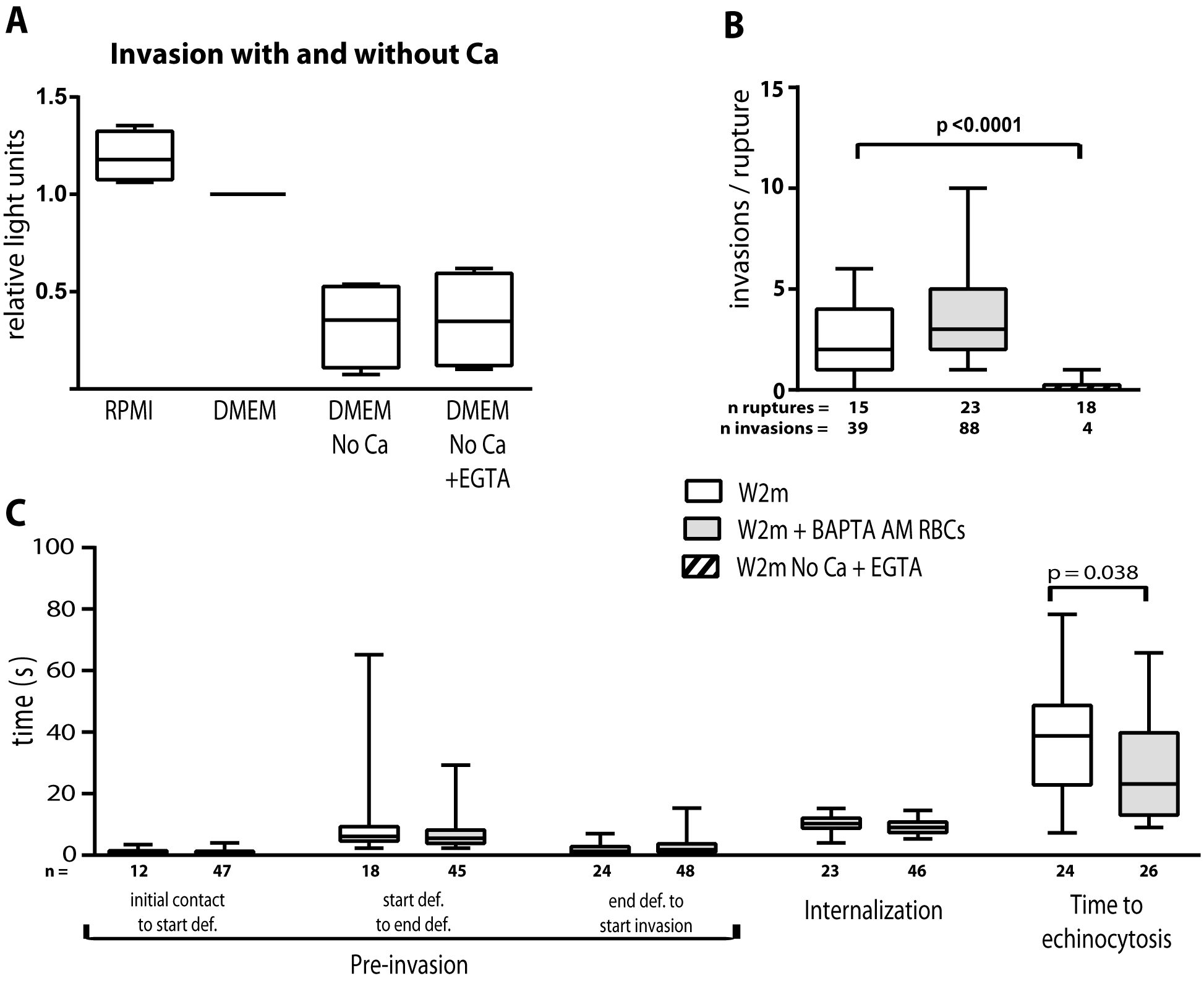

Supplement: S1 Fig — (A) Invasion assay in normal (RPMI and DMEM) and calcium free (DMEM no Ca2+ and DMEM No Ca2+ +EGTA) media indicate that merozoites need calcium for efficient invasion. Late schizont-stage parasites expressing Nanoluciferase were incubated with erythrocytes in the various media for 90 minutes-/+ heparin at 100μg/mL. After this the unruptured schizonts and merozoites were destroyed by sorbitol treatment and the new ring-stage parasites resulting from successful invasions were grown were for 72 hrs in complete RPMI media. The quantity of parasites was measured by assaying their luciferase levels in relative light units (RLU). To remove background levels of breakthough parasites ie, those parasites that had already invaded prior to the start of the experiment or had not been removed by sorbitol treatment, the RLU of heparin was subtracted from the untreated RLU. The median RLU of DMEM was normalised to 1.0 and the RLU of the other media relative to this are shown. The data represent four separate experiments each performed with triplicate samples. (B) Video microscopy of untreated parasites (W2m) and parasites whose erythrocyte hosts had been treated with BAPTA-AM indicate that chelation of Ca2+ introduced during invasion did not reduce the invasion rate in terms of invasion per schizont rupture. In contrast, treatment with Ca2+ free media +EGTA, caused the number of invasions per schizont rupture to significantly decline. (C) Comparison between the timings of W2mef invasion steps into BAPTA-AM treated and untreated erythrocytes indicates chelation of erythrocyte calcium has no major impact except for slightly decreasing the time to echinocytosis. For all figures, the horizontal line denotes the median, the box denotes the 25th to 75th percentiles and the whiskers the total data range. (TIF) [file ppat.1004670.s003.tif]
